# Supplementary material for: Evaluation of 3-Deoxy-D-Arabino-Heptulosonate 7-Phosphate Synthase (DAHPS) as a Vulnerable Target in Mycobacterium tuberculosis
Source: Microbiol Spectr. 2022 Jul 14;10(4):e00728-22. doi: 10.1128/spectrum.00728-22 (PMC9430761; doi:10.1128/spectrum.00728-22)
Supplement: Supplemental file 1 — Supplemental material. Download spectrum.00728-22-s0001.pdf, PDF file, 0.8 MB [file spectrum.00728-22-s0001.pdf]

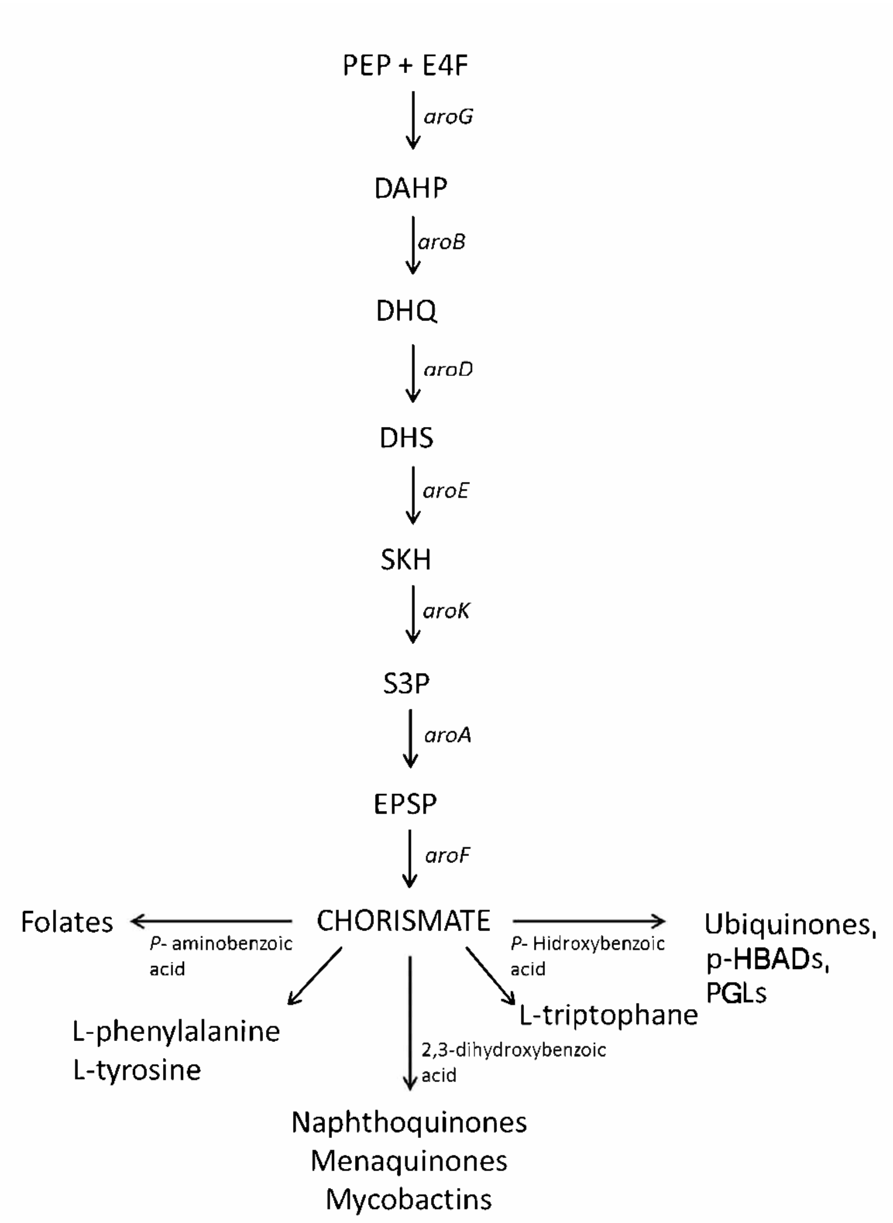

Figure S1: Schematic of the shikimate pathway and additional terminal pathways leading to the aromatic compounds. PEP: phosphoenolpyruvate; E4: D-erythrose 4-phosphate; *aroG*: gene encoding DAHP synthase; DAHP: 3-deoxy-d-arabino-heptulosonate-7-phosphate; *aroB*: gene encoding DHQ synthase; DHQ: 3-dehydroquinate; *aroD*: gene encoding DHQ dehydratase; DHS: 3-dehydroshikimate; *aroE*: gene encoding SKH dehydrogenase (SDH); SKH: shikimate; *aroK*: gene encoding SKH kinase; S3P: shikimate- 3-phosphate; *aroA*: gene encoding EPSP synthase; EPSP: 5-enolpyruvylshikimate-3-phosphate; *aroF*: gene encoding chorismate synthase.

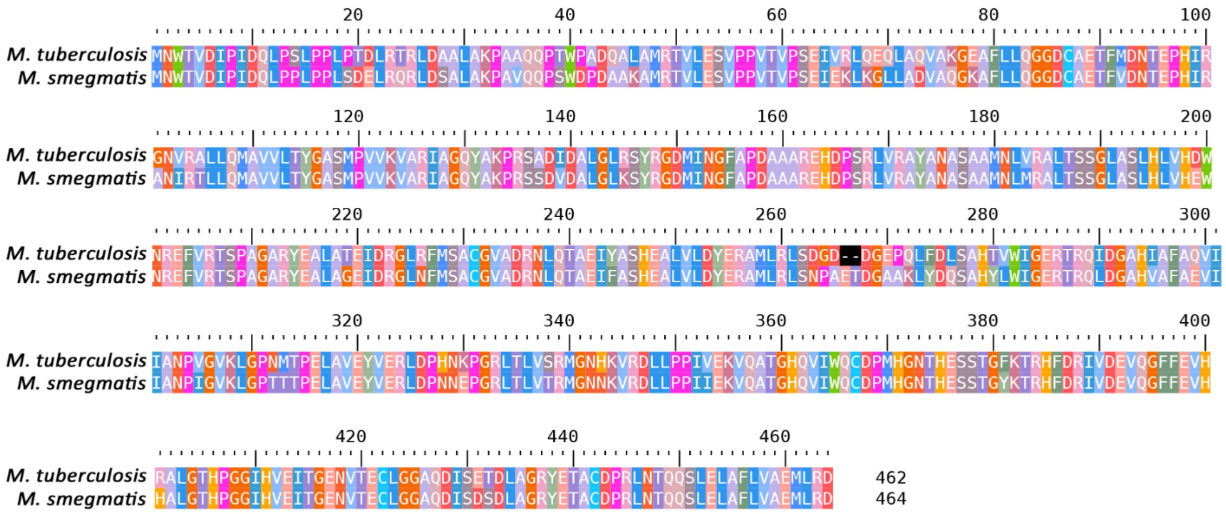

Figure S2: Pairwise alignment of aroG from M. tuberculosis and MSMEG_4244 from M. smegmatis. Identity: 405/464 (87.3%). Similarity: 436/464 (94.0%). Gaps: 2/464 (0.4%). Alignment parameters: Matrix: EBLOSUM62; Gap penalty: 10.0; Extend penalty: 0.5.

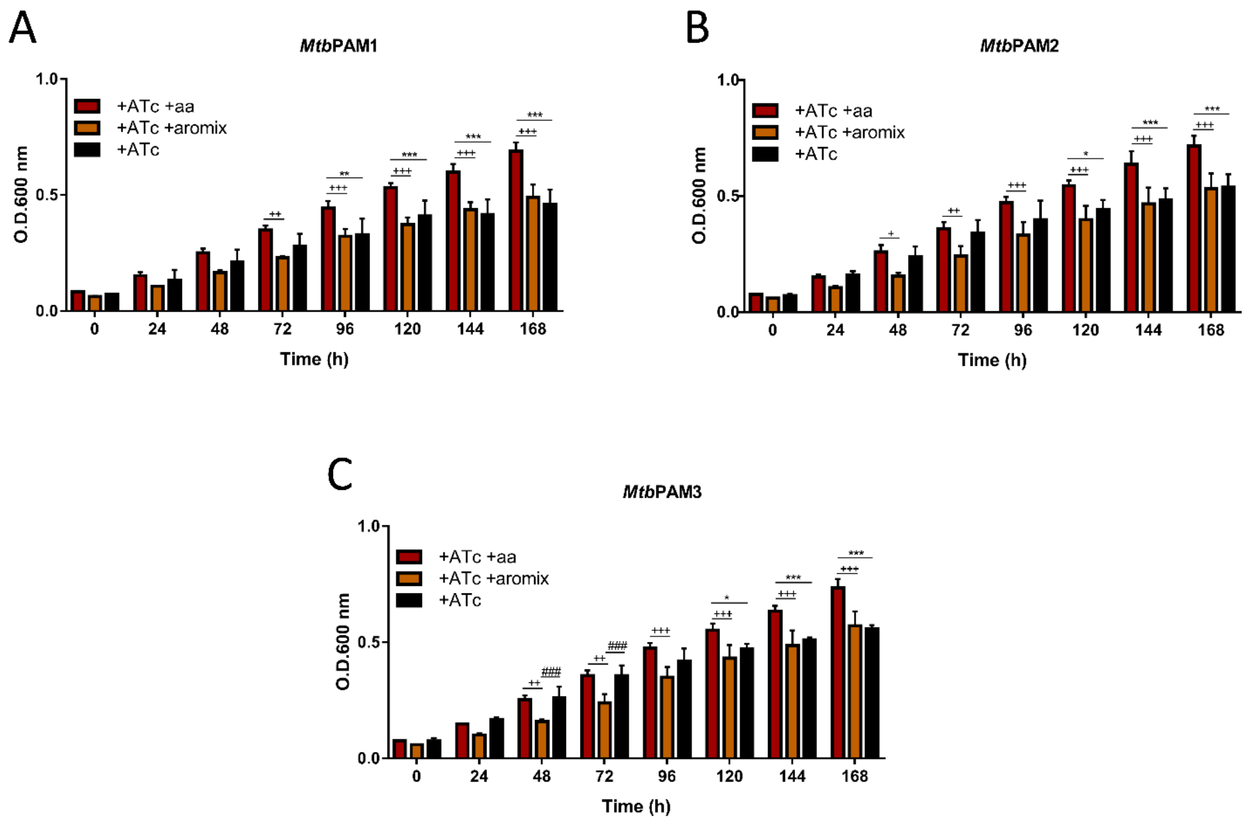

Figure S3: **Comparative effect of *aroG* silencing on *M. tuberculosis.*** Comparison of *aroG*-silencing cultures growth in presence of ATc 100ug/mL in medium supplemented with amino acids or aromix knockdown directed to PAM1 (A), PAM2 (B) or PAM3 (C). Mean ± standard deviation from biological and technical duplicates. For *=P value <0.03, **=P value <0.002 and ***=P value <0.001.

Table S1 - qQT-PCR primers and probes used in this study.

Target	Primer Forward	Primer Revese	Probe
**Rv2178c**	5’ctacagaccgccgaaatctatg 3’	5’ gtgcggacaggtcaaaca 3’	/56-FAM/catgttgag/ Zen/gtgtccgacggtga/3IABkFQ/
**MSMEG_4244**	5’ ggtacaagaccaggcacttc 3’	5’ cgagacattcggtgacgttt 3’	/56-FAM/tcgacgagg/ Zen/tgcagggcttt/3IABkFQ/
**SigA (*M. tuberculosis*)**	5’ catggtcgaggtgatcaacaa 3’	5’ gggtgatgtccatctctttgg 3’	/56-FAM/cattcaacg/ Zen/cgagctgctgca/3IABkFQ/
**SigA (*M. smegmatis*)**	5’ cgagaagttcgactacaccaag 3’	5’ ccatgtgcaccgggatac 3’	/56-FAM/tggcgtagg/Zen/ tcgagaacttgtagc/3IABkFQ/
**inhA**	5' gcattcgattgggttcatgc 3'	5’ atacgaatacgccgagatgtg 3’	56-FAM/atgggcatc/Zen/aacccgt tcttcga/3IABkFQ/
**mmpL3**	5’ ctggtcaacgtcgtgatgaa 3’	5’ ggcaggtatttctcgctgatac 3’	/56-FAM/tggtggtga/Zen/tggtgct gctgat/3IABkFQ/

Table S2 – Essentiality and vulnerability of *aro* genes from *M. tuberculosis* and *M. smegmatis*.

M. tuberculosis				*M. smegmatis*			
gene	Rv number	Essentiality[1]	VI[2]	gene	MSMEG number	Essentiality[3]	VI[2]
aroG	Rv2178c	essential	-13.36	*aroG*	MSMEG_4244	uncertain	-0.2
aroB	Rv2538c	essential	-9.2	*aroB*	MSMEG_3033	uncertain	-4.93
aroD	Rv2537c	essential	-5.92	*aroQ*	MSMEG_1922	uncertain	-3.52
				aroQ	MSMEG_2532	uncertain	0.11
aroE	Rv2552c	NonEssential	-9.01	*aroE*	MSMEG_3028	uncertain	-4.02
aroK	Rv2539c	Essential	-10.16	*aroK*	MSMEG_0453	uncertain	0.24
				aroK	MSMEG_3031	uncertain	-7.18
aroA	Rv3227	Essential	-9.00	*aroA*	MSMEG_1890	uncertain	-7.56
aroF/aroC	Rv2540c	Essential	-11.38	*aroC*	MSMEG_3030	uncertain	-9.05

[1]TnSeq essentiality predictions for *M. tuberculosis* taken from DeJesus et al. 2017 (PMID: 28096490).
[2]VI: Vulnerability Index. Vulnerability predictions taken from Bosch & DeJesus et al. Cell. 2021 (PMID: 34297925).
[3]TnSeq essentiality predictions for *M. smegmatis* taken from Dragset et al. 2019 (PMID: 31388080). Data obtained from https://pebble.rockefeller.edu.
